# Good news

**DOI:** 10.1590/S1516-31802006000100001

**Published:** 2006-01-05

**Authors:** Álvaro Nagib Atallah

After years of effort, finally the Cochrane Library has been included in the Institute for Scientific Information (ISI) database. So, from now on it will be possible to access the Cochrane Library impact factor, which is defined by the number of citations over a two-year period, divided by the total number of published papers in each Journal. This is a simplistic but practical way of evaluating the work of researchers in many countries.

Usually the quality of a published paper is evaluated by the database in which the journal was published, while assuming that it is difficult to publish in many journals that belong to the ISI database. However, recent comparison of the quality of randomized clinical trials in the five journals with the highest impact factor in the field of orthopedics and sports medicine that are included in the ISI database showed that, although the quality of randomized clinical trials most frequently is higher in the ISI than when they are published only in Medline, the proportion of papers considered to be good quality within the ISI setting of journals was about 40%. In other words, 60% of the papers published in the five top journals in the field have considerable flaws. So the quality of each published paper must be critically appraised individually, and there are lots of things to be done regarding the methodology of clinical trials, even in ISI journals.

Recently, a new way of evaluating medical journals was applied to the 120 top impact-factor ISI journals. McKibbon et al.^1^ (2004) evaluated these journals by looking at the "number needed to read" (NNR) index. In other words, how many papers need to be read in each "top" journal to find one paper that would be considered relevant to the reader and that was also of high methodological quality. Well done! The answer depends on the field of knowledge, but in general internal medicine, 95% of the Cochrane Library reviews were classified as good quality and relevant, and the NNR was 1.1. Among the top medical journals in terms of impact factor, New England Journal of Medicine and Lancet had an NNR of about 20 papers that needed to be read to be able to classify one as relevant and having good methodology.

It might be said that these authors were more interested in systematic reviews. But this is a major interest of medical journal readers worldwide. Fortunately, the Cochrane Library takes great methodological care and publishes systematic reviews that are considered to be the best level of evidence for decision-making. Probably when the impact factor is calculated for the Cochrane Library, we will realize that we have a journal with high impact factor and a low "number needed to read" and both evaluation tendencies will be satisfied.
